# Chronic Lateral Ankle Instability Repair Technique Using All-Inside and All-Knotless Soft-Tissue Anchors

**DOI:** 10.1016/j.eats.2025.103471

**Published:** 2025-02-22

**Authors:** Bruno S. Pereira, Maria Inês Peixoto, Luís Fabião, Nuno Esteves, Guilherme França, Filipe Sá Malheiro, João Espregueira-Mendes

**Affiliations:** a Clínica Espregueira–FIFA Medical Centre of Excellence, Porto, Portugal; b Unidade Local de Saúde de Barcelos/Esposende, Barcelos, Portugal; c ICVS/3B’s–PT Government Associate Laboratory, Braga, Portugal; d Foot and Ankle Unit, Human Anatomy Unit, School of Medicine, University of Barcelona, Barcelona, Spain; e Dom Henrique Research Centre, Porto, Portugal; f School of Medicine, University of Minho, Braga, Portugal; g Unidade Local de Saúde de Barcelos/Esposende, Braga, Portugal; h 3B’s Research Group–Biomaterials, Biodegradables and Biomimetics, University of Minho, Headquarters of the European Institute of Excellence on Tissue Engineering and Regenerative Medicine, Guimarães, Portugal

## Abstract

Chronic lateral ankle instability (CLAI) is common in sports. Whereas traditional open Broström-Gould surgery remains the gold standard for anterior talofibular ligament repair, advances in arthroscopic techniques with knotless suture anchors offer promising alternatives, reducing recovery time and complications. This article presents a minimally invasive arthroscopic technique for anterior talofibular ligament reconstruction with knotless anchors, reattaching the ligament to the fibula and minimizing risks of knot irritation and impingement. Postoperatively, patients follow a structured rehabilitation protocol, returning to full activity by 12 weeks. Compared with open techniques, this approach yields superior functional outcomes and esthetic benefits and addresses intra-articular issues associated with CLAI. The knotless arthroscopic technique yields a stable ligament repair with fewer neurologic risks, enhancing patient satisfaction and success. This procedure presents a safe, effective alternative for CLAI treatment.

The ankle sprain is the most common ligament injury in sports, but it also affects the general population.^1^ In 85% of all lateral ankle sprains, the anterior talofibular ligament (ATFL) is the injured ligament.^2^ Although most ankle sprains progress to complete functional recovery, up to 70% of patients continue to experience chronic symptoms and limitations,^3^^,^^4^ potentially leading to chronic lateral ankle instability (CLAI).

In recent times, there has been a growing interest in using arthroscopy to treat CLAI, especially using suture anchors.^5^ The Broström arthroscopic procedure has shown positive clinical results.^6^ This procedure involves surgically separating the ATFL from the capsule and reattaching it to the fibula using knotted or knotless anchors.^6^^,^^7^ It is important to acknowledge that there is still an overall rate of complications (e.g., knot irritation^5^) of 11.6%,^6^ which is similar to the rate with open techniques. To simplify the arthroscopic process, a knotless suture anchor was developed.^8^ Despite the Broström-Gould surgical procedure being the gold-standard technique,^9^ some studies have shown that arthroscopic techniques yield better clinical and functional results; however, few have used knotless anchors. Given these recent advancements, we present a surgical technique of ATFL reconstruction using knotless anchors for CLAI, aiming to offer a more anatomically precise, minimally invasive, and safer reconstruction approach.

## Surgical Technique

### Preoperative Planning and Patient Setup

The surgical decision is made after a careful imaging evaluation of the tibiotarsal joint (magnetic resonance imaging and radiography examinations) in conjunction with physical examination findings and a clinical history suggestive of CLAI. The choice of anesthesia is made by the patient, anesthesiologist, and surgeon, taking into account the different factors influencing the preferred anesthesia technique, such as patient comorbidities.

**Fig 1:**
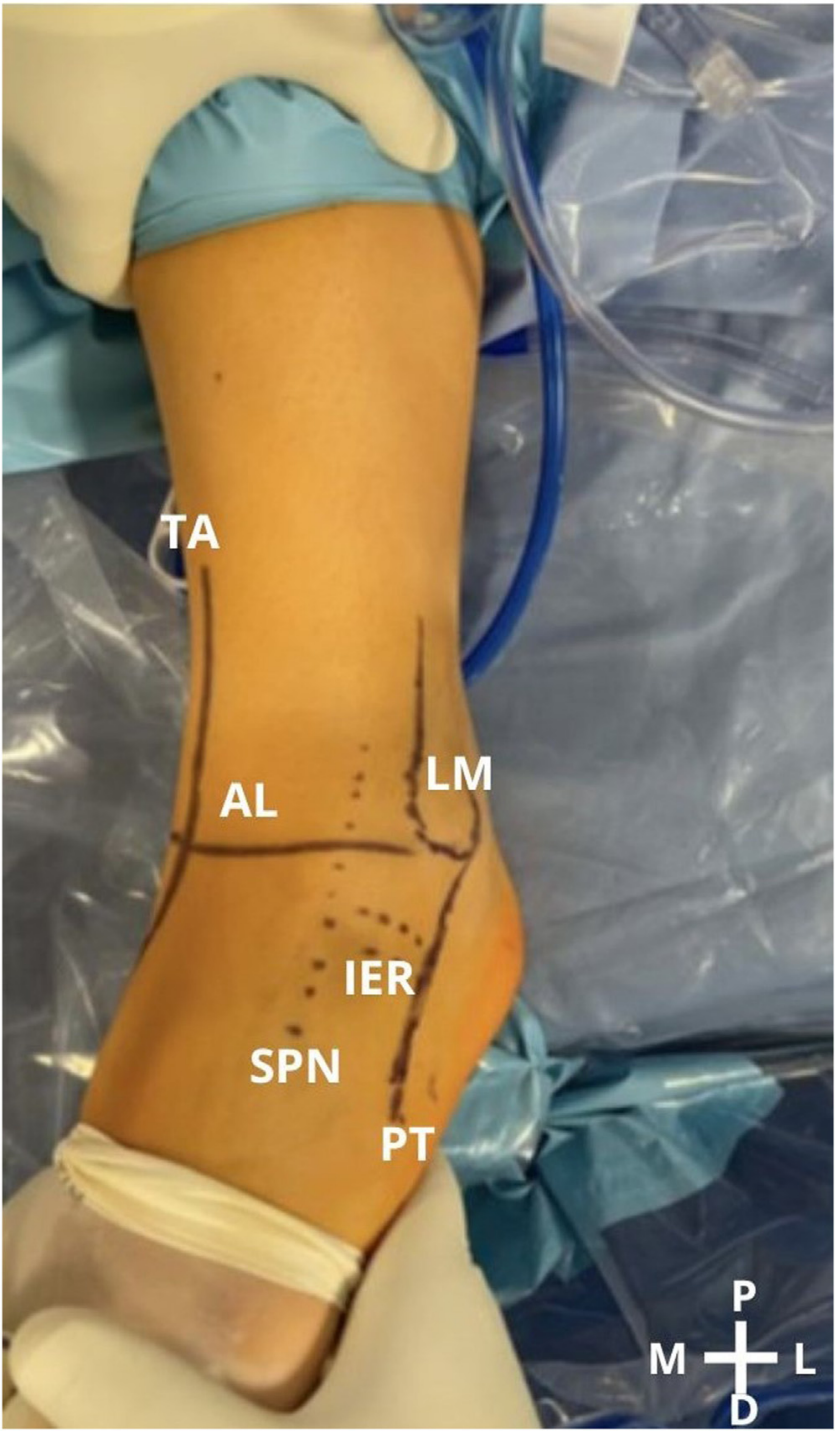
A left ankle is shown with the patient supine. Reference lines and points are drawn to help to create the arthroscopic portals. (AL, articular line; D, distal; IER, inferior extensor retinaculum; L, lateral; LM, lateral malleolus; M, medial; P, proximal; PT, peroneal tendon; SPN, superficial peroneal nerve; TA, tibialis anterior tendon.)

The patient is positioned in the supine position with a tourniquet on the proximal thigh (Video 1). To check instability, the anterior drawer test is performed, and thereafter, the limb is disinfected (Table 1). By use of a marker, anatomic reference lines and points are drawn on the skin to help create the classic anterior arthroscopy portals. The anterior tibial tendon is identified during ankle dorsiflexion and is placed as laterally as possible (Table 2). The medial malleolus, lateral malleolus, superficial peroneal nerve, and articular line are also located (Fig 1). With these steps, it becomes possible to create the anterior portals. The anteromedial portal is created first, followed by the anterolateral portal, with direct visualization.

**Table 1:** Key Points of Surgical Procedure

Position the patient supine with a tourniquet at the proximal thigh.
Identify the surgical landmarks on the skin.
Perform placement of the anterior medial portal, followed by the lateral portal, with direct visualization.
Perform an inspection from medial to lateral to evaluate the joint and the tissue to be repaired, remove damaged tissue, and prepare the footprints of the ligamentous structures on the fibula—ATFL and CFL.
Place the FiberTak guide and slide the guidewire drill down (1.6 mm for soft bone or 1.8 mm for hard bone) 2 times.
Insert the anchor through the drill guide and impact with hammer.
Remove the black rubber ring that secures the wires; next, remove the blue wire with the needle and cut the needle; and finally, remove the guide and anchor introducer. Slowly pull on the wires to open the intraosseous anchor and secure it.
Place the wire blue tip on the DX Mini Scorpion, and with the DX Mini Scorpion inverted, pass it through the ligament. Outside the cannula, pass the blue wire through the looped end of the black-and-white FiberLink loop to the dark mark on the blue wire.
With a series of light pulls on the FiberLink loop, pull the blue wire inside the anchor while simultaneously pulling the ligament into the footprint, with the foot in a neutral position.
Cut the wire with arthroscopic scissors.
Repeat the procedure with the second anchor.
Check the tension of the ligament’s reinsertion, and perform the anterior and anterolateral drawer tests to verify joint stability.

ATFL, anterior talofibular ligament; CFL, calcaneofibular ligament.

**Table 2:** Pearls and Pitfalls

Pearls	Pitfalls
Correct identification of the anterior tibial tendon, medial malleolus, lateral malleolus, superficial peroneal nerve, and articular line is important to perform the arthroscopic portals.	Not recognizing other anatomic predisposing factors for CLAI may lead to potential failure.
The surgeon should carefully remove damaged tissue and prepare the attachment site to receive the anchorage.	When preparing the footprint with a shaver, the surgeon must be careful not to remove all of the cortical bone.
Correct identification of the footprints of ATFL and CFL is necessary.	A bone tunnel should be created 1 cm from the distal tip of the fibula.
Reattach the tissue that is compatible with the ligament.	The remaining ligament should be grabbed with a grasper, to check if the tissue is of good quality.
For soft bone, a 1.6-mm drill should be used, whereas for hard bone, a 1.8-mm drill should be used.	The surgeon must be careful not to damage the superficial peroneal nerve, when performing the anterolateral portal.

ATFL, anterior talofibular ligament; CFL, calcaneofibular ligament; CLAI, chronic lateral ankle instability.

### Surgical Approach

**Fig 2:**
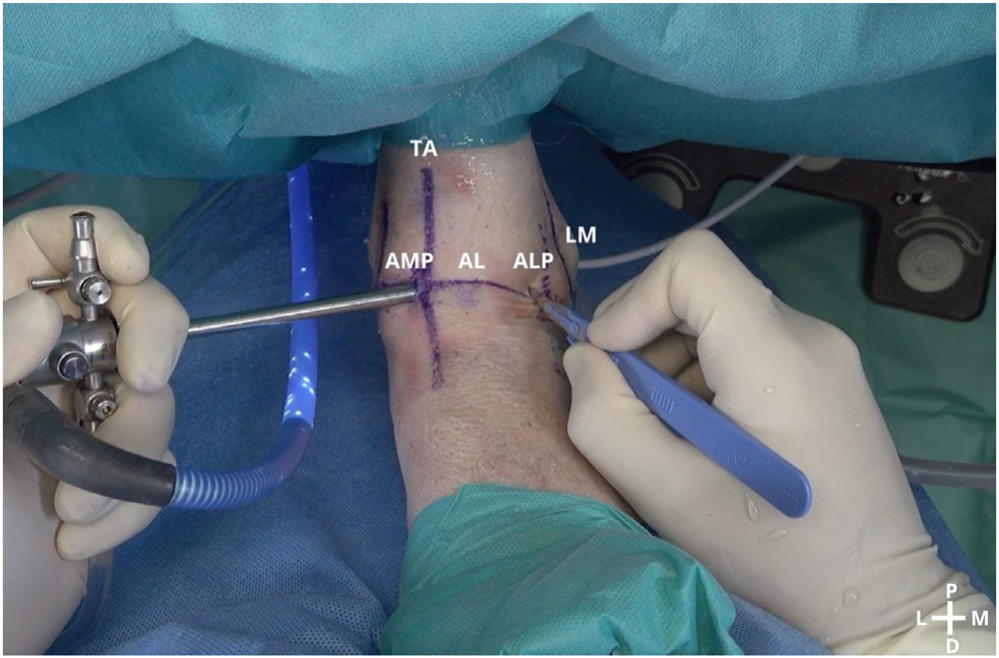
A left ankle is shown with the patient supine. A conventional arthroscopic anteromedial portal (AMP) is established, and the incision for the anterolateral portal (ALP) is made. (AL, articular line; D, distal; L, lateral; LM, lateral malleolus; M, medial; P, proximal; TA, tibialis anterior tendon.)

We use the standard anteromedial and anterolateral portals for ankle arthroscopy (Fig 2). The anteromedial portal is established just medial to the tibialis anterior tendon at the level of the ankle joint. Palpation of the anterior joint line is performed to identify the optimal entry point, ensuring avoidance of neurovascular structures. A small incision is made through the skin, and blunt dissection is used to reach the joint capsule. Under direct visualization, the portal is introduced using a trocar and cannula to access the joint. The anterolateral portal is created lateral to the peroneus tertius tendon, at the same level as the anteromedial portal. Care is taken to avoid the superficial peroneal nerve, which is palpated and marked preoperatively. Similar to the anteromedial portal, a skin incision is made and blunt dissection is used to safely penetrate the joint capsule before inserting the trocar and cannula.

**Fig 3:**
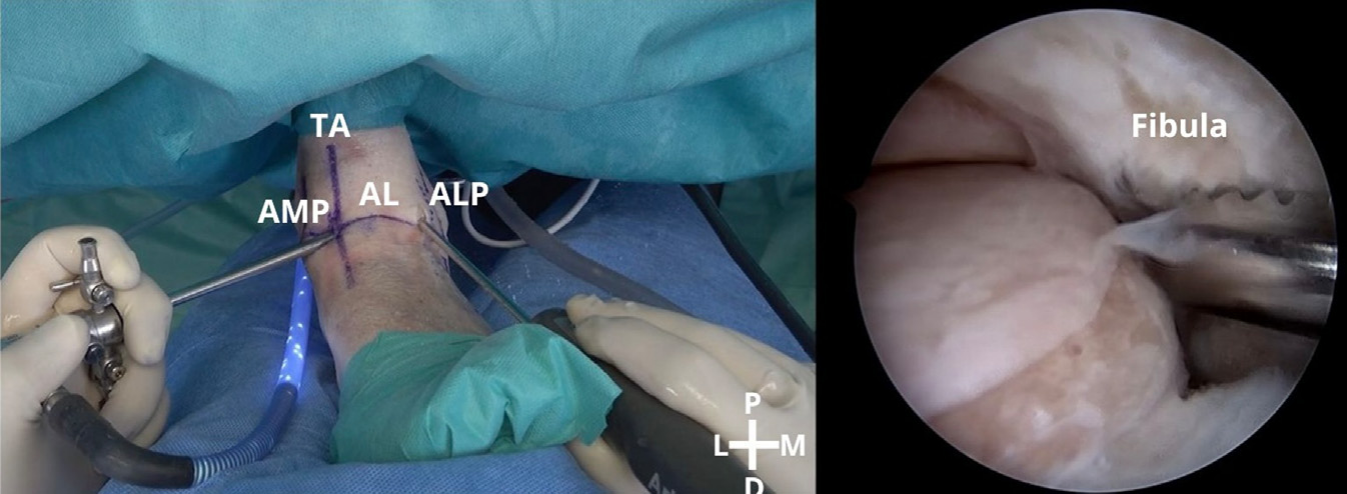
A left ankle is shown with the patient supine. The footprints of the ligamentous structures on the fibula are prepared. (AL, articular line; ALP, anterolateral portal; AMP, anteromedial portal; D, distal; L, lateral; M, medial; P, proximal; TA, tibialis anterior tendon.)

After creation of the 2 classic anterior portals, a medial-to-lateral inspection is conducted with the arthroscope to evaluate the joint in terms of cartilage, impingements, and ligamentous structures. Then, it is important to assess the tissue to be repaired, and the tissue is prepared to receive the anchorage. This step is crucial to remove the damaged tissue and prepare the footprints of the ligamentous structures on the fibula—ATFL and calcaneofibular ligament—using a shaver (Fig 3).

**Fig 4:**
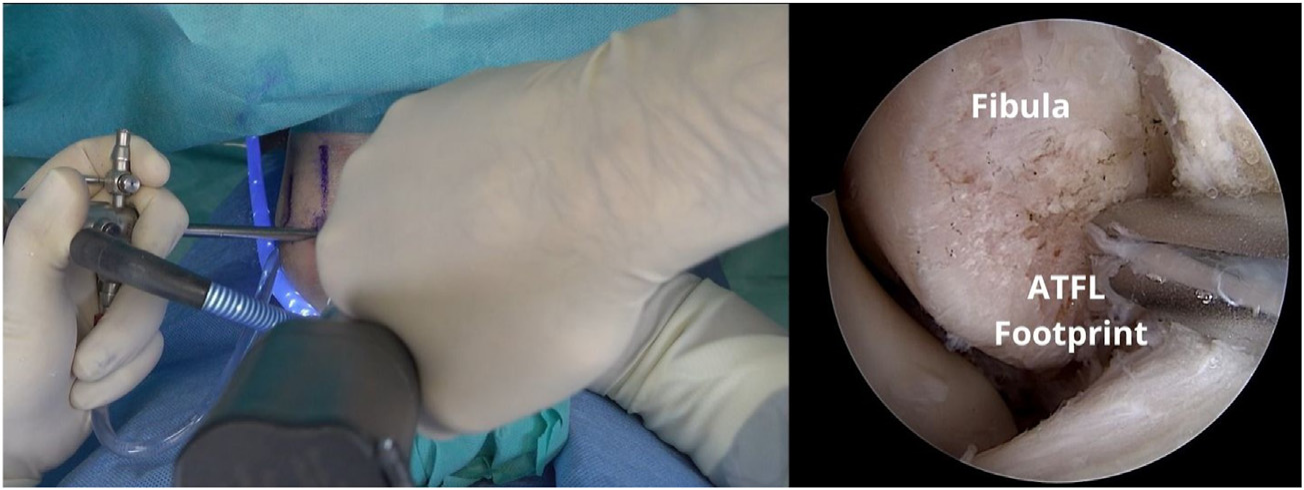
A left ankle is shown with the patient supine. The FiberTak guide is placed, and a bone socket is created, 1 cm from the distal tip of the fibula, where the anterior talofibular ligament (ATFL) footprint is located.

**Fig 5:**
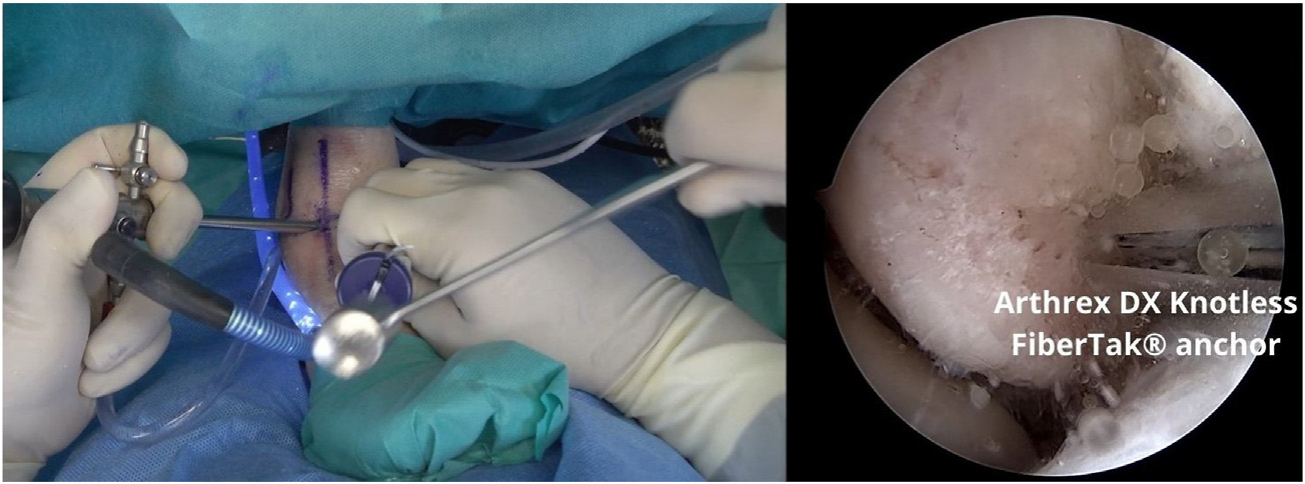
A left ankle is shown with the patient supine. Keeping the guide in place, the anchor (DX Knotless FiberTak anchor) is inserted and impacted with hammer.

The FiberTak guide (Arthrex) is placed, and a bone tunnel is created, 1 cm from the distal tip of the fibula, where the ATFL footprint is located, by sliding the guidewire to create a bone tunnel (Fig 4). For soft bone, a 1.6-mm guidewire is used, whereas for hard bone, a 1.8-mm guidewire is used. The drill is passed 2 times in the footprint to prepare the anchorage. Keeping the guide in place, the anchor (Arthrex DX Knotless FiberTak anchor) is inserted and impacted with gentle taps until the handle is flush with the guide (Fig 5).

**Fig 6:**
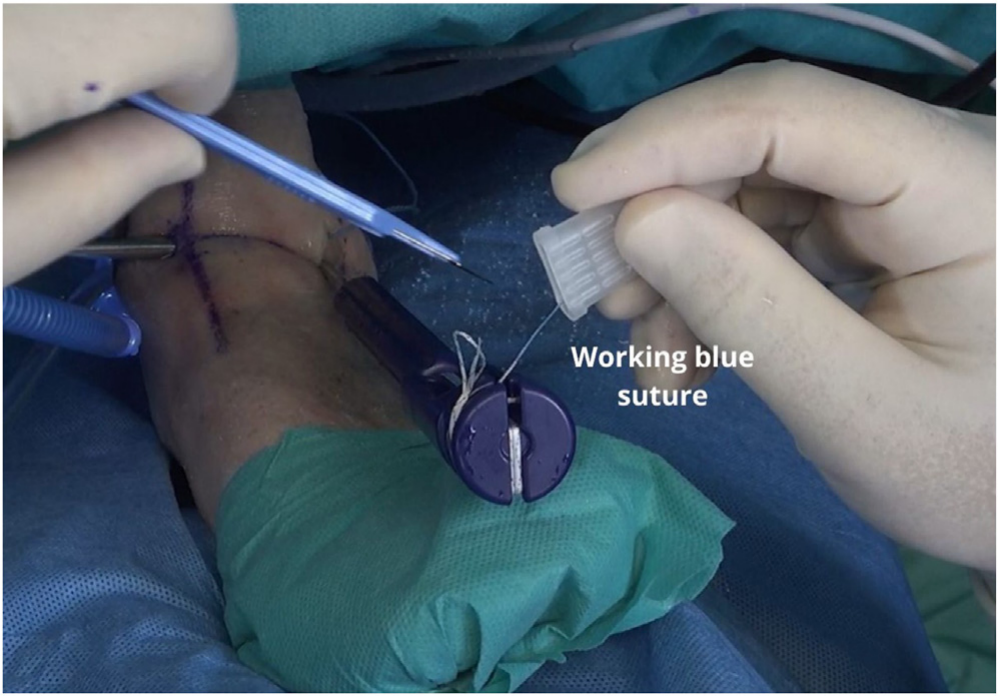
A left ankle is shown with the patient supine. The working blue suture with the needle is removed, and the needle is cut.

**Fig 7:**
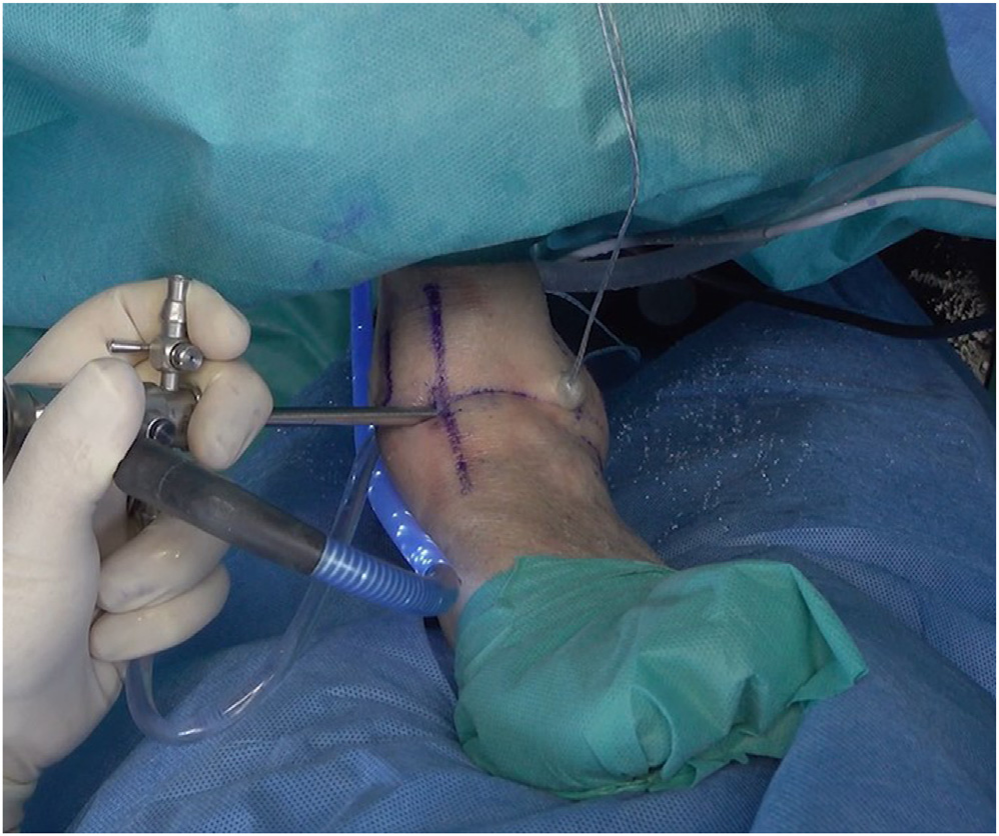
A left ankle is shown with the patient supine. The guide and the anchor introducer are removed.

**Fig 8:**
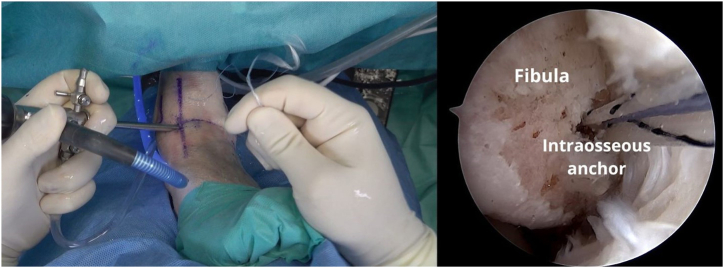
A left ankle is shown with the patient supine. The wires are slowly pulled on to open the intraosseous anchor and secure it.

The black rubber ring that secures the wires to release the sutures from the handle is removed. The working blue suture with the needle is removed (Fig 6). The guide and the anchor introducer are removed (Fig 7). Thereafter, the wires are slowly pulled on to open the intraosseous anchor and secure it (Fig 8).

**Fig 9:**
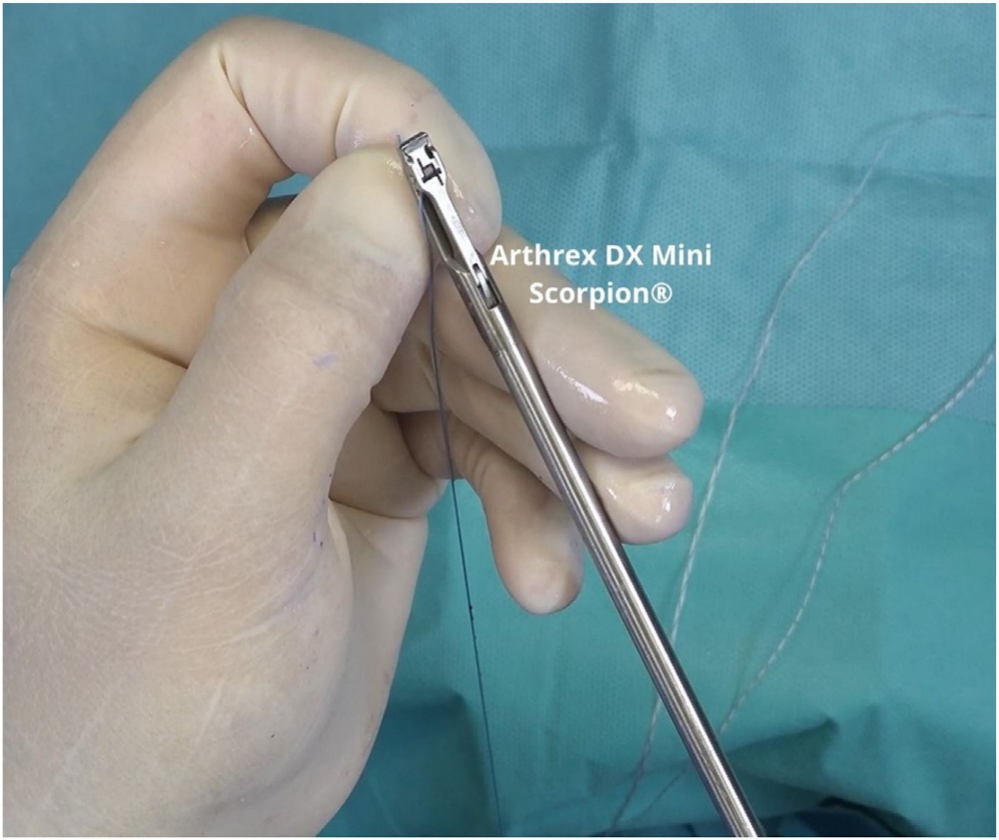
The working blue suture tip is placed on the DX Mini Scorpion.

**Fig 10:**
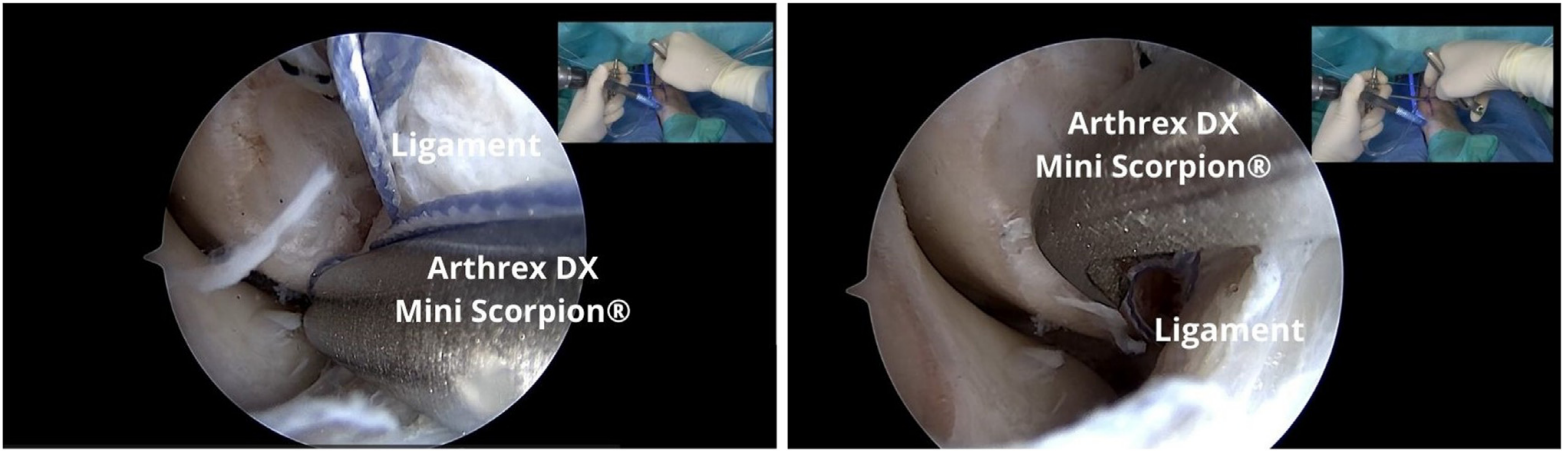
A left ankle is shown with the patient supine. The DX Mini Scorpion is inverted, and it is passed through the ligament.

**Fig 11:**
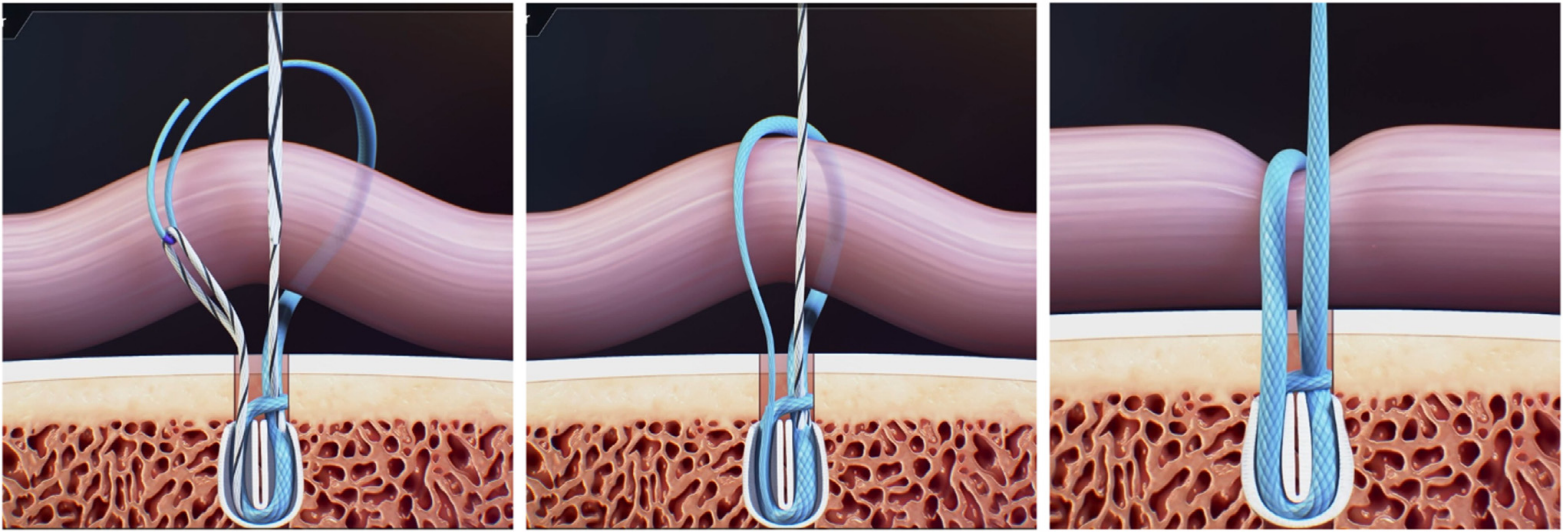
Knotless system.

**Fig 12:**
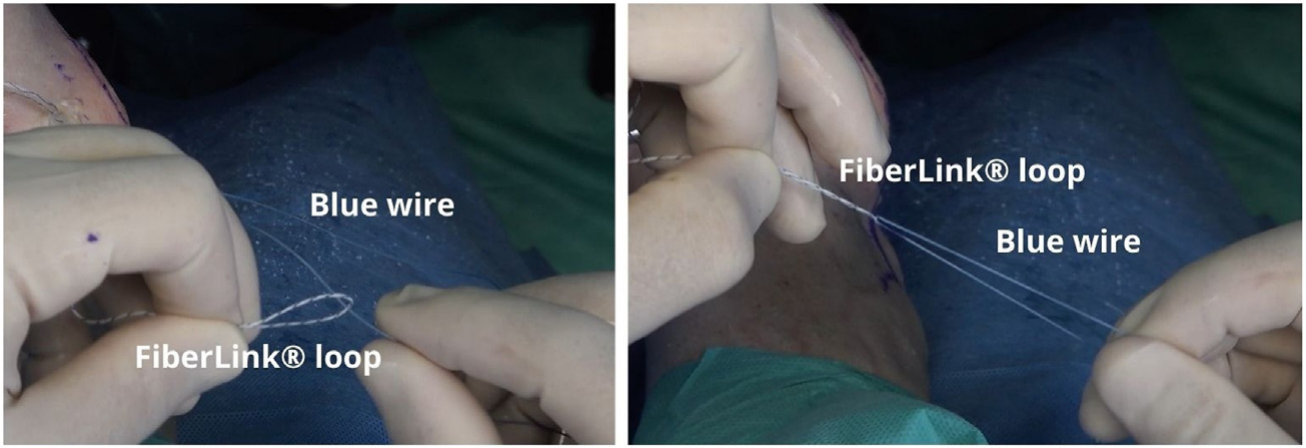
Outside the cannula, the blue wire is passed through the looped end of the black-and-white FiberLink loop to the purple mark on the blue wire.

The working blue suture tip is placed (Fig 9) on the Arthrex DX Mini Scorpion, and with the DX Mini Scorpion inverted, it is passed through the middle of the ligament to pass in an inside-out manner (Fig 10). The next step is based on the work principles of the anchor knotless system (Fig 11). Outside the cannula, the blue wire is passed through the looped end of the black-and-white FiberLink loop (Arthrex), which is white with black stripes, to the purple mark on the blue wire (Fig 12).

**Fig 13:**
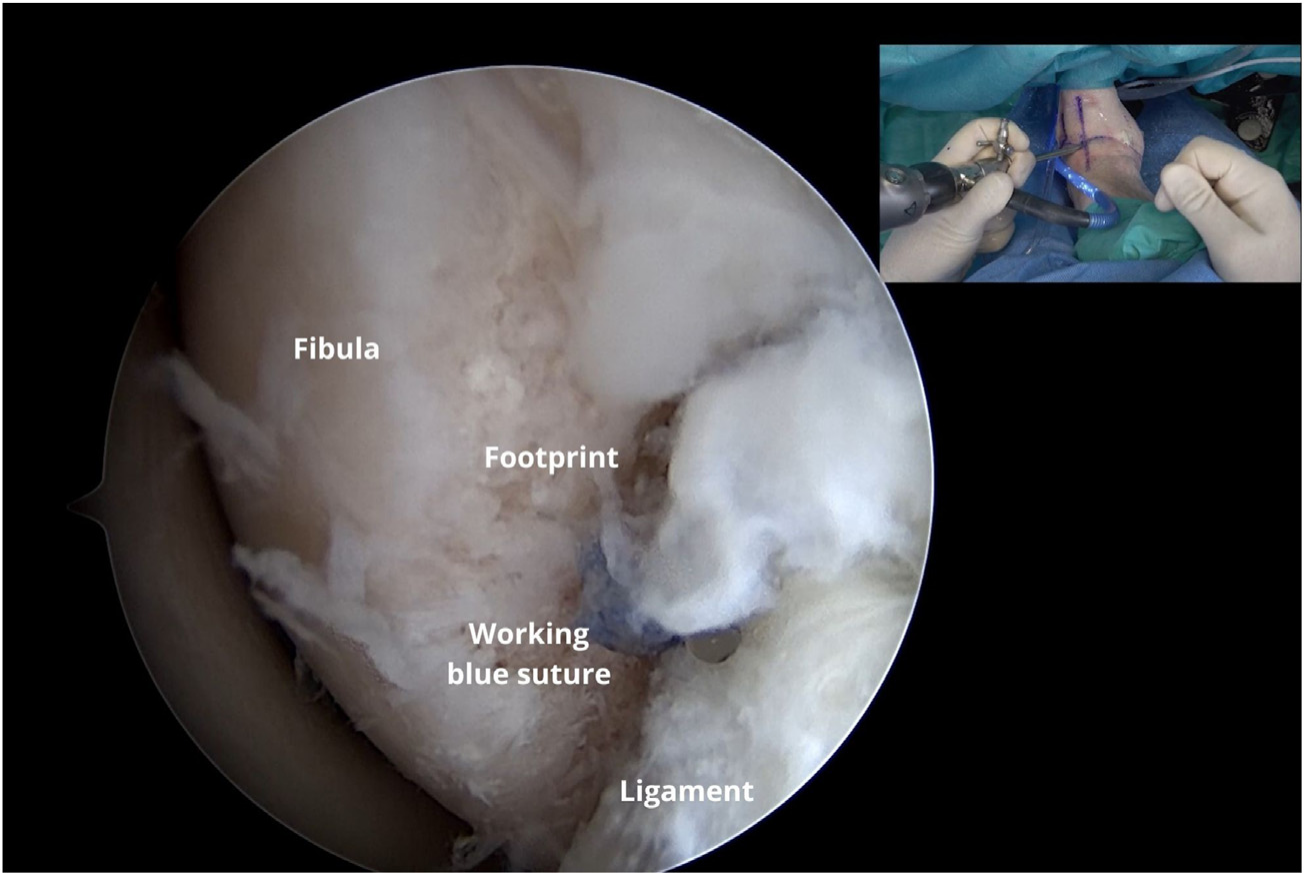
A left ankle is shown with the patient supine. The working blue suture is inside the anchor and the ligament is pulled to the footprint, with the foot in a neutral position.

With a series of light pulls on the FiberLink loop, the working blue suture is pulled inside the anchor while the ligament is simultaneously being pulled into the footprint, with the foot in a neutral position (Fig 13). The wire is cut with arthroscopic scissors. The procedure is repeated with the second anchor.

With this type of anchor, we do not make sliding knots and, thus, there is less risk of soft-tissue impingement occurring when tensioning the ligament. Because of this, we can tension the ligament more effectively.

**Fig 14:**
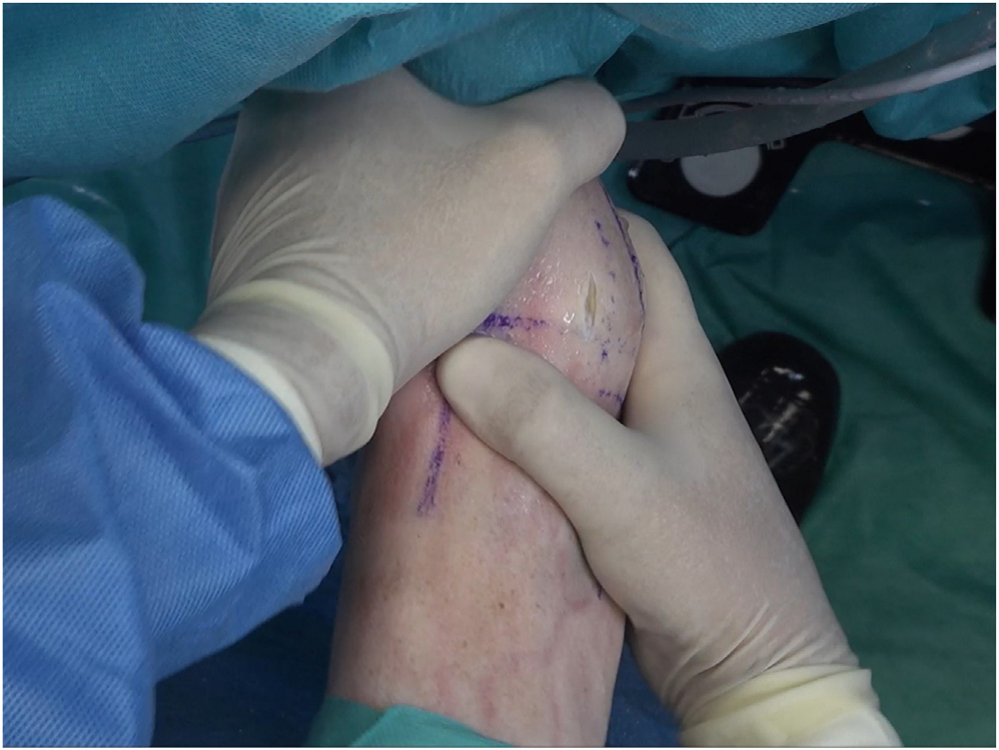
A left ankle is shown with the patient supine. The anterolateral drawer test is performed to confirm joint stability.

The ligament’s reinsertion tension should be checked to ensure that the ligament is properly anchored and not lax. This is confirmed by applying traction to the ligament, and anterior and anterolateral drawer tests should be performed to confirm joint stability (Fig 14).

### Postoperative Rehabilitation

After the surgical procedure, load cast immobilization is performed for 2 weeks, followed by use of a walker boot; full weight bearing is allowed according to tolerance with the aid of crutches. After 2 weeks, the patient is allowed to start active ankle movements. From 4 to 6 weeks, the walker boot continues to be used. The patient can walk at full weight without the aid of crutches. From 6 weeks onward, walking is performed without a boot and with full weight bearing, and the use of crutches may be necessary initially. Resistance exercises should be carried out with a gradual increase in strength (open and closed kinetic chain activities, as well as functional activities). Return to sport is expected at 12 weeks, with full recovery at 4 months.

## Discussion

Several techniques for the treatment of CLAI have been developed over the years, and multiple options are described in the literature.^6^ Open Broström-Gould surgery has been the established standard for many years. The emergence of arthroscopy has prompted exploration for a less invasive option, aiming to minimize postoperative pain and shorten recovery time.^10^ Arthroscopic repair involves surgically separating the ATFL from the capsule and reattaching it to the fibula using anchors. Instead of exposing the distal fibula through an open incision, the surgeon inserts the anchors while viewing the distal fibula through an arthroscope.^7^ This procedure has shown positive clinical results, including minimized postoperative pain, smaller scars, reduced swelling, less disturbance of cutaneous sensation, and shortened recovery time.^6^^,^^7^^,^^10^ The most commonly reported complications are neuritis of the superficial peroneal or sural nerve and pain or discomfort caused by a prominent anchor or suture knot.^11^

An arthroscopic approach for repairing the ATFL using knotless suture anchors successfully allows the restoration of the ATFL’s stability (Table 3) while avoiding neurologic issues or major complications.^11^ Beyond the esthetic advantages, the arthroscopic procedure has the added benefit of diagnosing and treating associated intra-articular issues most effectively. Intra-articular problems are frequently linked to ankle instability, and addressing them during surgery is crucial for achieving the best results.^12^ By creating a bone tunnel, this technique allows for the removal of a minimal amount of bone. The fact that no sliding knots are made reduces the risk of soft-tissue impingement occurring when tensioning the ligament, and thus, the ligament can be tensioned more effectively. It has been shown that knotless anchor fixation exhibits greater strength and less variability compared with knotted constructs in securing a suture loop.^13^ Because a knotted suture system is not used, knot-related pain is expected to be eliminated, thereby improving patient outcomes. All these points support the adoption of arthroscopic repair with knotless suture anchors.

**Table 3:** Advantages, Risks, and Limitations of Technique

Advantages
The technique allows restoration of the ATFL’s stability.
To create a bone tunnel, minimal bone is removed.
The fact that no sliding knots are made reduces the risk of soft-tissue impingement occurring when tensioning the ligament, and thus, the ligament can be tensioned more effectively.
The technique can be performed simultaneously with other bony procedures.
Risk and limitations
The surgeon must be careful not to damage the superficial peroneal nerve.

ATFL, anterior talofibular ligament.

A potential limitation of this technique is related to the risk of iatrogenic damage to the superficial peroneal nerve, which is influenced by the surgeon’s experience. An important step is to create a bone tunnel 1 cm from the distal end of the fibula to maintain the anatomic insertion of the ATFL.

In conclusion, we present an all-inside technique for the treatment of CLAI using knotless suture anchors, avoiding the risks of knotted sutures. It is a simple, effective, and secure technique, providing an inconspicuous repair without knot impingement or loosening and with minimal bone removal.

## Disclosures

All authors (B.S.P., M.I.P., L.F., N.E., G.F., F.S.M., J.E-M.) declare that they have no known competing financial interests or personal relationships that could have appeared to influence the work reported in this paper.

## Supplementary Data

Video 1 Surgical technique for chronic lateral ankle instability. The patient is placed supine with a tourniquet at the proximal thigh. Anatomic reference lines and points are drawn on the skin to help create the classic anterior arthroscopy portals. After the 2 portal incisions are made, a medial-to-lateral inspection is conducted with the arthroscope to evaluate the joint in terms of cartilage, conflicts, and ligamentous structures. Then, it is important to assess the tissue to be repaired. Next, the tissue is prepared to receive the anchorage. Through this, we prepare the footprints of the ligamentous structures on the fibula—anterior talofibular ligament (ATFL) and calcaneofibular ligament (CFL). The FiberTak guide is placed, and a bone socket is created, 1 cm from the distal tip of the fibula, by sliding the guidewire to create a bone tunnel. With the guide kept in place, the anchor is inserted and impacted with gentle mallet taps until the handle is flush with the guide. The working blue suture with the needle is removed, and the needle is cut. Next, the guide and anchor introducer are removed. Thereafter, the wires are slowly pulled on to open the intraosseous anchor and secure it. Posteriorly, the working blue suture tip is placed on the DX Mini Scorpion, and with the DX Mini Scorpion inverted, it is passed through the ligament. The next step is based on the work principles of the anchor knotless system. Outside the cannula, the blue wire is passed through the looped end of the black-and-white loop, which is white with black stripes, to the purple mark on the blue wire. Thereafter, with a series of light tugs on the loop, the working blue suture is pulled inside the anchor while the ligament is simultaneously being pulled into the footprint, with the foot in a neutral position. Finally, the wire is cut with arthroscopic scissors. The procedure is repeated with the second anchor.
